# A Theoretical Model for Debonding Prediction in the RC Beams Externally Strengthened with Steel Strip and Inorganic Matrix

**DOI:** 10.3390/ma14174961

**Published:** 2021-08-31

**Authors:** Francesco Bencardino, Mattia Nisticò

**Affiliations:** Department of Civil Engineering, University of Calabria, Via P. Bucci, Cubo 39B, Rende, 87036 Cosenza, Italy; mattia.nistico@unical.it

**Keywords:** debonding, fabric reinforced cementitious matrix, theoretical analysis, reinforced concrete, repair, steel reinforced geopolymeric matrix, steel reinforced grout, strengthening

## Abstract

This paper shows a theoretical model for predicting the moment–curvature/load–deflection relationships and debonding failure of reinforced concrete (RC) beams externally strengthened with steel reinforced geopolymeric matrix (SRGM) or steel reinforced grout (SRG) systems. Force equilibrium and strain compatibility equations for a beam section divided into several segments are numerically solved using non-linear behaviour of concrete and internal steel bars. The deflection is then obtained from the flexural stiffness at a mid-span section. Considering the appropriate SRGM-concrete bond–slip law, calibrated on single-lap shear bond tests, both end and intermediate debonding failures are analysed. To predict the end debonding, an anchorage strength model is adopted. To predict intermediate debonding, at each pair of flexural cracks a shear stress limitation is placed at concrete–matrix interface and the differential problem is solved at steel strip–matrix interface. Based on the theoretical predictions, the comparisons with experimental data show that the proposed model can accurately predict the structural response of SRGM/SRG strengthened RC beams. It can be a useful tool for evaluating the behaviour of externally strengthened RC beams, avoiding experimental tests.

## 1. Introduction

In the last four decades, the need for repair and rehabilitation of existing reinforced concrete (RC) structures has become an increasingly important challenge for civil engineering community. Among the various methods developed, externally bonded-fibre reinforced polymer (EB-FRP) systems have been widely accepted as an effective and suitable system for the strengthening and retrofitting of RC members [1,2,3]. EB-FRP systems offer several benefits, including freedom from corrosion, excellent weight to strength ratio, good fatigue resistance, flexibility to conform to any shape, and broad application [4,5,6,7,8,9]. Despite their satisfactory performance, FRP composite materials have several drawbacks including high cost, low fire resistance, poor sustainability, and low compatibility with traditional building materials. Promising newly developed inorganic matrices potentially represent a cost-effective and durable alternative to epoxy resins used for FRP systems [10,11,12,13,14,15,16,17,18,19,20,21,22,23,24]. Different inorganic-based systems for strengthening of RC structures have been proposed: textile reinforced concrete (TRC) [10,11], textile reinforced mortar (TRM) [12,13], fabric reinforced cementitious matrix (FRCM) [14,15], steel reinforced geopolymeric matrix (SRGM) [16,17,18,19], and steel reinforced grout (SRG) [20,21,22,23,24]. Specifically, TRC consists of multiaxial textile fabrics bonded to concrete surfaces with a fine-grained, high strength concrete; TRM uses textile fabrics and polymer-modified mortar as a bonding agent; FRCM consists of unidirectional or bidirectional fabrics made of traditional (carbon, glass, aramid) or innovative (basalt, poliparafenilenbenzobisoxazolo) fibres bonded to concrete surfaces with an inorganic matrix, whereas SRGM and SRG systems comprise unidirectional steel strips embedded into inorganic matrices.

Within the broad category of inorganic composite materials, polymer-based inorganic systems (SRGM) have received growing interest in recent years. The experimental investigations on the flexural strengthening of RC beams by Menna et al. [19], Katakalos and Papakonstantinou [18], Vasconcelos et al. [25], and Bencardino and Condello [16,17] are pointed out. Menna et al. [19] concluded that the beams strengthened with carbon fabrics bonded to concrete surfaces using a geopolymeric matrix provided a negligible increase in the bending capacity due to poor adhesion between the carbon fabric and the geopolymeric matrix. However, the size of the carbon fibres used was designed to achieve optimal adhesion with epoxy resin. Katakalos and Papakonstantinou [18] observed that no delamination of steel strips due to fatigue loading occurred for sixteen RC beams externally strengthened with high strength steel strips impregnated in an inorganic geopolymeric matrix. Vasconcelos et al. [25] investigated the use of metakaolin based geopolymers mortars as a repairing layer or binding agent to ensure strong adhesion between externally bonded carbon sheets and the concrete substrate. Recently, Bencardino and Condello [16,17] investigated a new potential structural application of the inorganic matrices. Specifically, the matrix was used at the same time as a corrosion inhibitor of the bottom internal steel bars, repairing/restoring of the cover layer, and a binding agent to apply stainless steel strengthening strips. The proposed solution, an alternative to the EB technique, was conceived for upgrading RC structures with extensively deteriorated cover concrete and/or corroded steel bars. It includes three operations in one: inhibiting–repairing–strengthening (IRS), and consists of the installation of the SRGM system during the repairing/restoring of the cover concrete (IRS-SRGM system). The IRS-SRGM solution reduces the time and cost of strengthening interventions compared with the EB technique. Test results showed that the IRS-SRGM system provides good structural performance, increasing the load carrying capacity up to 30% with a limited reduction of ductility compared to the RC control beam. Different failure modes were observed during the tests compared with the EB-FRP systems. Consequently, to theoretically predict the behaviour of RC members strengthened with steel strips and inorganic matrices, appropriate analytical models and/or bond–slip laws are required.

The bond behaviour between the external strengthening system and the concrete substrate is an issue that concerns and generally controls the ultimate capacity of strengthened members. It is affected by a high level of uncertainty due to the complex interaction of several phenomena, such as concrete cracking, steel yielding in longitudinal bars, interface adhesion properties, the type of components and the matrix composition. As a result of this partial understanding, different analytical approaches have been proposed within the scientific literature for FRP systems and adopted by the most common codes, standards and guidelines to perform the required safety checks [1,2,3]. However, proper standard documents or guidelines for the design of EB/IRS inorganic composite systems are not yet available. The American Concrete Institute (ACI) proposed a specific guideline for fabric reinforced cementitious matrix (FRCM) systems [26], but no bond–slip laws and analytical models to predict end debonding and intermediate debonding were provided. It should be noted that for inorganic-based systems, the debonding phenomenon is more complex than in FRP systems due to matrix cracking as well as interlaminar debonding that could occur at the fibre–matrix interface [14,15,16,17,23,27]. Nevertheless, the reliability and adaptability of some analytical models and bond–slip laws proposed for the FRP systems to predict the behaviour of RC beams externally strengthened with inorganic-based systems were recently analysed [28,29].

In the present study, a theoretical procedure was developed for the prediction of the moment–curvature relationship and, hence, the load carrying capacity and deflections of RC beams strengthened with steel strips and inorganic matrices (SRGM/SRG systems). In the proposed procedure, a sectional analysis is firstly carried out, where the cross-section of strengthened RC member is divided into a number of concrete segments. The model is able to predict both the end debonding in the un-cracked regions, and intermediate debonding at SRGM–concrete and fibre–matrix interfaces in the cracked regions of the strengthened beams. To this end, an appropriate bond–slip law, calibrated on SRGM–concrete shear bond tests, was used in the numerical model. Finally, to check the reliability of the model experimental and theoretical comparisons were carried out.

## 2. Theoretical Modelling

In this section, the theoretical procedure is presented. Specifically, the constitutive law of materials, the evaluation of the moment–curvature/load–displacement curves and the end/intermediate debonding predictions are described. Computer code using MATLAB R2020a [30] has been developed for implementing the model.

Similar numerical techniques were used by [31,32,33] for response prediction of reinforced concrete (RC) beams strengthened with prestressed near-surface-mounted FRP reinforcements or hybrid FRP/steel reinforcements. However, in these studies debonding failure was not taken into account.

### 2.1. Stress–Strain Material Models

**Figure 1:**
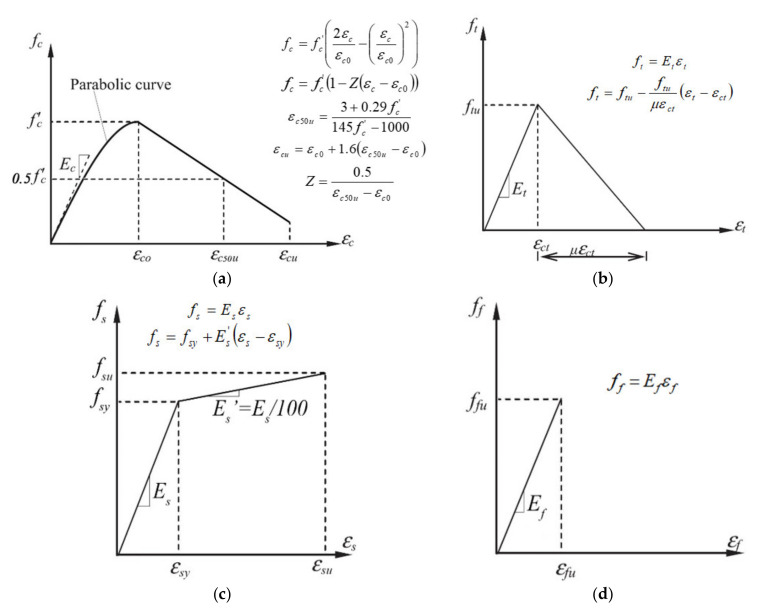
Stress–strain relationships: (**a**) concrete in compression; (**b**) concrete in tension; (**c**) internal steel reinforcement; (**d**) external strengthening system.

The well-known stress–strain relationships of concrete, internal steel, and external reinforcement implemented in the model are showed in Figure 1. Specifically, the stress–strain law proposed by Kent and Park [34] for confined concrete in compression is given in Figure 1a.

A bi-linear stress–strain relationship (Figure 1b) is used to model the concrete in tension. To model the internal steel, both in compression and tension, a bi-linear stress–strain relationship with hardening is used (Figure 1c).

Finally, the stress–strain relationship of external strengthening system (Figure 1d) is assumed linear elastic up to failure.

In Figure 1a, *f_c_* and *ε_c_* are the compressive stress and strain of the concrete, respectively; *f_c_’* is the cylinder compressive strength; *E_c_* is the initial tangent modulus; *ε_c0_* (=0.002) is the strain in concrete at maximum stress; *ε_c50u_* is the strain corresponding to 0.5 *f_c_’*; *ε_cu_* is the ultimate strain of concrete; and *Z* is a factor controlling the rate of compressive strength decay.

In Figure 1b, *f_t_* and *ε_t_* are the tensile stress and strain of the concrete, respectively; *f_tu_* and *ε_ct_* are the tensile strength and corresponding tensile strain, respectively; *E_t_* is the tensile modulus, assumed to be the same as *E_c_*; and *μ* is a factor controlling the rate of tensile strength decay. The tension stiffening effect is taken into account in the model.

In Figure 1c, *f_s_* and *ε_s_* are the stress and strain of the internal steel bars, respectively; *f_sy_* and *ε_sy_* are the yield strength and corresponding strain, respectively; *f_su_* and *ε_su_* are the ultimate strength and corresponding strain, respectively; *E_s_* (=210,000 MPa) is the elastic modulus; and *E_s_’* (=*E_s_*/100) is the hardening modulus.

Finally, in Figure 1d, *f_f_* and *ε_f_* are the stress and strain of the reinforcing strip, respectively; *E_f_* is the elastic modulus; *f_fu_* and *ε_fu_* are the ultimate tensile strength and strain, respectively.

### 2.2. Moment–Curvature/Load–Deflection Relationships

**Figure 2:**
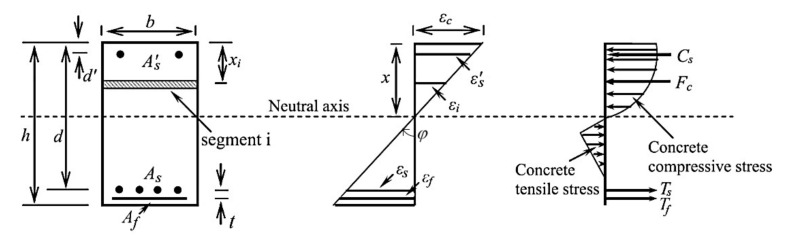
Strains, stresses, and forces of an RC section strengthened with the IRS-SRGM system.

Figure 2 illustrates an RC section externally strengthened with the IRS-SRGM system, that is divided into a number, *n*, of segments. The analysis starts by assuming a small value of strain at the concrete extreme compression fibre. For each strain *ε_c_* at the top level of concrete section, the neutral axis depth (*x*) is iteratively obtained when equilibrium of the forces is satisfied. According to the assumption that the section remains plane after bending, the strain in each compressive or tensile concrete segment can be expressed as:

(1) $$\varepsilon_{i}=\frac{x-x_{i}}{x}\varepsilon_{c}\;$$

where *ε_c_* is the strain at the top compression level and *ε_i_* is the concrete compressive or tensile strain at mid-depth of *i*^th^ segment. Assuming perfect bonds between all materials the strains can also be obtained as follows:

(2) $$\frac{\varepsilon_{c}}{x}=\frac{\varepsilon_{s}^{\prime }}{x-d^{\prime }}=\frac{\varepsilon_{s}}{x-d}=\frac{\varepsilon_{f}}{x-\left(d+t\right)}$$

where *d*′ and *d* are the top and bottom steel reinforcement depths, respectively; *t* is the distance between bottom internal steel and external reinforcement, that is equal to *d′*/2 and *d′* for the IRS and EB strengthening techniques, respectively.

The total concrete force includes the contribution of compressive and tensile concrete and is calculated by the following relationship:

(3) $$F_{c}=\sum_{i=1}^{n}f_{ci}h_{i}b\;$$

where *f_ci_* is the concrete compressive or tensile stress at the centroid of the *i*^th^ segment, *h_i_* (=*h/n*) is the thickness of the *i*^th^ segment and *b* is the beam width. This summation extends over all compressive and tensile segments of concrete section. The forces in the top steel bars, bottom steel bars and external strengthening strip can also be calculated as:

(4) $$C_{s}=A_{s}^{\prime }f_{s}^{\prime },\;T_{s}=A_{s}f_{s},\;T_{f}=A_{f}f_{f}\;$$

where *f_s_′*, *f_s_*, and *f_f_* are the stresses in top steel, bottom steel, and external strip, respectively. By considering the equilibrium of forces, the neutral axis depth *x* is the only unknown parameter, and it can be determined.

The bending moment *M* is then calculated by taking moments of internal forces about the neutral axis:

(5) $$M=\sum_{i=1}^{n}f_{ci}h_{i}b\left(x-x_{i}\right)+C_{s}\left(x-d^{\prime }\right)+T_{s}\left(x-d\right)+T_{f}\left(x-\left(d+t\right)\right)$$

The beam curvature *φ* can also be determined from the strain distribution as follows (Figure 2):

(6) $$\phi =\frac{\varepsilon_{c}}{x}$$

In order to obtain the load–midspan deflection curve, the flexural stiffness (*EI_eff_*) of member at the location of the maximum moment is firstly determined from the moment-curvature relationship at each loading as:

(7) $$EI_{eff}=\frac{M}{\phi }$$

The midspan deflection (Δ) is then calculated using the elastic deflection formula of simply supported beams loaded with two-point loads, each P/2:

(8) $$\Delta =\frac{0.5Pa\left(3L^{2}-4a^{2}\right)}{24EI_{eff}}$$

where *a* is the shear span and *L* is the span length. The strain in the concrete extreme compression fibre of the section is incrementally increased and the above procedure is iteratively repeated for each value of strain. The analysis is stopped when either the tensile strain in the external reinforcing system reaches the ultimate strain (*ε_f_* = *ε_fu_*) or the concrete strain in the extreme compression fibre reaches the ultimate compressive strain (*ε_c_ = ε_cu_*).

In the theoretical procedure presented above, the full composite action is assumed and the debonding failures are not taken into account. The debonding load is evaluated by means of calculations presented in the next sections.

### 2.3. Debonding Predictions

The general opinion among researchers is that the debonding failure is initiated by high interfacial shear and normal stresses at external strengthening–matrix or matrix–concrete interfaces, when the strength of the weakest element is exceeded [35,36,37].

In general, for externally strengthened RC members there are two types of debonding: end debonding and intermediate debonding [35,36]. End debonding is the failure that starts near the end-side of reinforcing strip and propagates either along the tensile steel reinforcement (end cover separation) or near the bond area (end interfacial delamination). The end cover separation is typical of FRP-strengthened members, but it was rarely observed in RC members strengthened in flexure with inorganic-based systems. In fact, experimental investigations highlighted that RC beams strengthened with EB-SRGM or similar systems, failed by brittle end debonding at the reinforcement–concrete interface and/or delamination at fibre–matrix interface without damage to the concrete substrate [16,17,19,23].

On the other hand, intermediate debonding failure starts either from a flexural crack (flexure crack delamination) or an inclined flexure-shear crack (shear crack delamination) and propagates to the end-side of the reinforcing strip. The flexural delamination occurs when large vertical cracks take place, whereas the shear delamination has been found to be the most critical due to the brittleness of failure [36]. However, for the RC beams with sufficient shear capacity, as is the case in this research, the shear delamination can be avoided and mode I propagation, due to normal stresses at interface, can be neglected [37]. In the proposed procedure, it was assumed that both end/intermediate debonding failures occur mainly due to mode II (shear stresses at the SRGM–concrete and steel strip–matrix interfaces).

**Figure 3:**
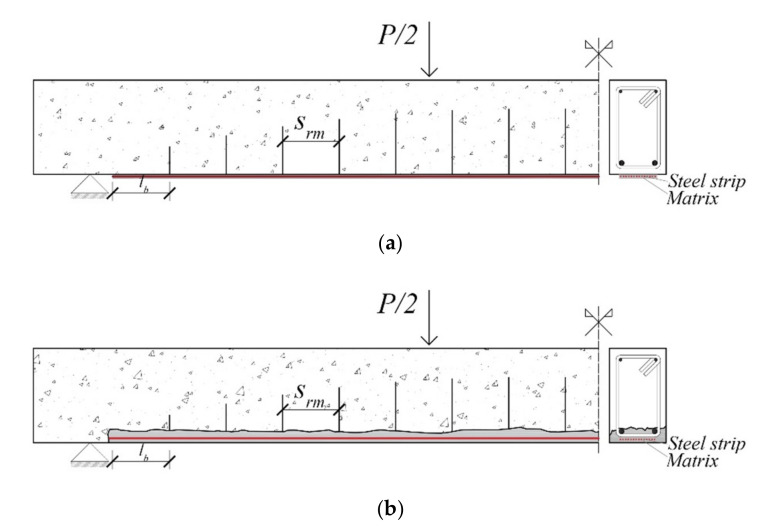
Strengthened RC beams: (**a**) EB technique; (**b**) IRS technique.

In order to evaluate the debonding load, an incremental procedure was developed. Figure 3a,b show, under cracked conditions, a half of RC beams strengthened with EB and IRS techniques, respectively. According to the experimental failure modes observed in SRGM/SRG-strengthened RC beams, the debonding failure occurred after yielding of tensile steel bars [16,17,19,21,22,23,24]. Consequently, the load was incrementally increased from yielding to failure, i.e., until the achievement of ultimate strain in compressive concrete or tensile steel strip. The procedure was stopped when a debonding criterion was satisfied. Both end and intermediate debonding at the concrete–matrix and steel strip–matrix interfaces are considered in the present analysis.

To simplify the calculations, a constant crack spacing (*s_rm_*) after yielding until failure, is assumed. In fact, it is well-known that few cracks appear after yielding thanks to the steel bars. Most cracks occur before yielding increasing their width until failure. According to Fib 14 [3], the crack spacing between two subsequent cracks can be calculated assuming constant average bond stresses of both internal (*τ_sm_*) and external (*τ_fm_*) reinforcements, as follows:

(9) $$\tau_{sm}=1.85f_{tu}$$

(10) $$\tau_{fm}=0.44f_{tu}$$

(11) $$s_{rm}=2\frac{M_{cr}}{z_{m}}\frac{1}{\tau_{fm}b_{f}+\sum_{i=1}^{h}\tau_{sm}d_{s}\pi }$$

where *M_cr_* is the cracking moment, calculated by cross-section analysis; *d_s_* is the diameter of *i*^th^ bottom steel bar; *h* is the number of bottom steel bars; and *z_m_* is the mean level arm determined taking into account the axial stiffness of different layers of reinforcement:

(12) $$z_{m}=0.85\frac{(d+t)E_{f}A_{f}+dE_{s}A_{s}}{E_{f}A_{f}+E_{s}A_{s}}$$

In order to take into account the additional tensile force in the longitudinal reinforcements due to shear stress, as suggested by Eurocode 2 [38], the shift rule of the bending moment diagram is applied.

#### 2.3.1. Interfacial Behaviour of SRGM System Bonded to Concrete

The load carrying capacity of externally strengthened RC members is affected by debonding of the external strengthening layer. In order to evaluate the interfacial bond failure mechanisms, an appropriate local bond–slip law (bond stress–slip relationship, *τ_bond_-s*) is required. To this purpose, twelve single-lap shear bond tests on SRGM–concrete joints by varying the bonded length (*l_b_* = 100, 150, 200, 250, 300, and 400 mm) were carried out [36]. Two specimens for each bonded length were tested. The concrete prisms were 150 mm wide × 200 mm deep × 600 mm long. An SRGM composite system of 50 mm width (*b_f_*) was bonded to the two faces of 150 mm × 600 mm. The mechanical properties of the concrete substrate were similar to those of the EB/IRS-SRGM strengthened RC beams briefly described in the next section [16,17]. From this study [39] an effective bonded length (*l_eff_*) of 200 mm was defined.

In order to calibrate an analytical bond–slip law for SRGM system, the approach proposed by Dai et al. [40] for FRP system was adopted. It assumes that, at any location of an FRP–concrete interface, under the boundary condition of zero free end slip (*l_b_ ≥ l_eff_* = 200 mm), exists a unique *τ_bond_–s* relationship and a unique relationship between the strain of FRP sheets (*ε_f_*) and interfacial slip (*s*) as expressed below:

(13) $$\varepsilon_{f}=A\left[1-\exp\left(-Bs\right)\right]$$

where *A* and *B* are two parameters evaluated by means of non-linear regression analysis. The mean values of *A* and *B*, calculated for the specimens with *l_b_ ≥ l_eff_*, were equal to 7.82‰ and 6.46 mm^−1^, respectively [39]. By considering Equation (13) and equilibrium at the interface, the following expressions for the bond–slip relationship (*τ_bond_-s*) and interfacial fracture energy (*G_f_*) can be derived:

(14) $$\tau_{bond}=A^{2}BE_{f}t_{f}\exp\left(-Bs\right)\left[1-\exp\left(-Bs\right)\right]$$

(15) $$G_{f}=0.5A^{2}E_{f}t_{f}$$

where *t_f_* is the equivalent thickness of external strengthening strip. By substituting the values of *A* and *B*, the expression of the bond–slip law and the value of fracture energy (0.54 N/mm) are obtained [39].

According to the failure mode detected for the single-lap shear bond tests [39], Equation (14) is used in the model to predict the intermediate debonding at steel strip–matrix interface of RC beams strengthened with the EB or IRS techniques. Furthermore, it should be noted that parameter *A* represents the analytical maximum strain of the stainless-steel strip for end debonding. This strain value was used in the proposed model to predict the end debonding failure of RC beams strengthened with either EB or IRS technique.

Further details regarding the test set-up of single-lap shear bond tests, experimental results and calibration of bond–slip laws are given in Bencardino et al. [39].

#### 2.3.2. End Debonding

In order to predict the end debonding failure mode, different approaches were proposed in the literature for FRP systems. The existing models can be generally classified into four categories, namely: shear capacity models [41], concrete tooth models [42], interfacial stress-based models [43] and anchorage strength models [2,3]. The shear capacity models [41] assume that the end debonding failure is related to the shear strength of concrete with or without the contribution of internal shear reinforcement. Concrete tooth models [42] make use of the concept of a concrete “tooth” between two adjacent cracks deforming like a cantilever at the base of the beam under action of horizontal shear stresses due to external reinforcement. In this case, debonding occurs when these shear stresses lead to tensile stresses at the root of the tooth that exceed the concrete tensile strength. The interfacial stress-based models [43] make use of interfacial stresses from an existing closed-form solution combined with a concrete failure criterion. It should be noted that the previous three models assume that the end debonding failure only depends on the mechanical properties of concrete substrate. However, this assumption is not appropriately consistent with the failure modes experimentally observed in RC beams strengthened with inorganic-based systems [14,15,16,17,23,27]. Consequently, these models need improvements for external strengthening systems with inorganic matrices.

**Figure 4:**
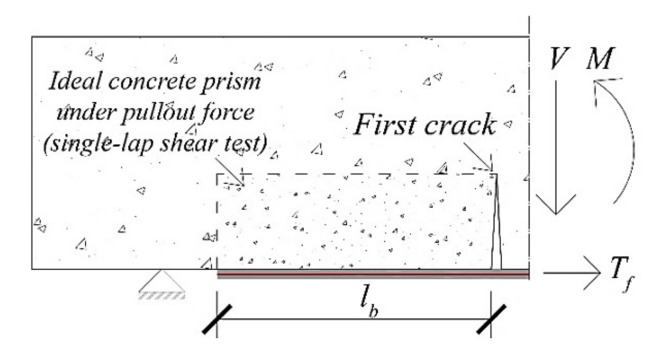
Anchorage strength model.

In this study, an anchorage strength model calibrated on single-lap shear bond tests is adopted. It assumes that, at the end uncracked regions of EB/IRS strengthened RC beams (Figure 4), the interface behaviour is similar to that of SRGM–concrete joints, presented in Section 2.3.1.

For *l_b_ ≥ l_eff_*, the maximum axial strain in strengthening strip for end debonding (*ε_f_,_ED_*), at first crack (see Figure 4), is equal to *A* (=7.82‰); whereas, for *l_b_ < l_eff_*, *ε_f,ED_* is reduced according to the following parabolic relationship suggested by CNR-DT200R1/2013 [2] and Fib 14 [3]:

(16) $$\varepsilon_{f,ED}=\frac{l_{b}}{l_{eff}}\left(2-\frac{l_{b}}{l_{eff}}\right)A$$

The first crack position along the beam, for each load step, is determined based on the cracking moment evaluated by means of the cross-section analysis presented in Section 2.2. It should be noted that only the first crack position varies during the incremental process, whereas the positions of the other cracks along the beam are fixed (Equation (11)). This assumption is required as the increase of external load and corresponding bending moment could reduce the length of the uncracked region (*l_b_*) and, consequently, the maximum axial strain in the reinforcing strip as obtained from Equation (16).

The safety factor for end debonding (*SF_ED_*), at each load step, is computed as follows:

(17) $$SF_{ED}=\frac{M_{Ed,ED}}{M_{Rd,ED}}\leq 1$$

where *M_Ed,ED_* and *M_Rd,ED_* (calculated assuming *ε_f_ = ε_f_,_ED_*) are the bending moment and bending strength at first crack, respectively. If *SF_ED_* is greater than one, end debonding failure occurs and the analysis is stopped.

#### 2.3.3. Intermediate Debonding

In order to predict the intermediate debonding failure mode, different approaches have been proposed in literature. The models can be classified by their approach to the problem as interfacial stress-based models [43], fracture mechanics-based models [17,37] and empirical/semi-empirical models [1,2,3]. Interfacial stress-based models [43] are the same as those used to predict end debonding (see Section 2.3.2). Fracture mechanics-based models [17,37] make use of elastic and fracture material properties to define a global debonding criterion in terms of energy dissipated at failure. The empirical/semi-empirical models provide simplified relations on a phenomenological basis to predict intermediate debonding failure without going into complex stress or fracture analyses. They are usually included in the design guidelines [1,2,3]. Specifically, two different approaches are used: design limitation on bond shear stress or design limitation on the tensile strain/stress in the external reinforcement.

In the proposed model, a combined criterion is used to predict intermediate debonding failures. Specifically, at each pair of flexural cracks (Figure 3), a shear stress limitation is placed at the concrete–matrix interface and the differential problem is solved at steel strip–matrix interface. In this way, the model is capable of predicting the two intermediate debonding failure modes observed during experimental tests, which are debonding at concrete–matrix and at reinforcing strip–matrix interfaces [16,17,21,22,23,24].

**Figure 5:**
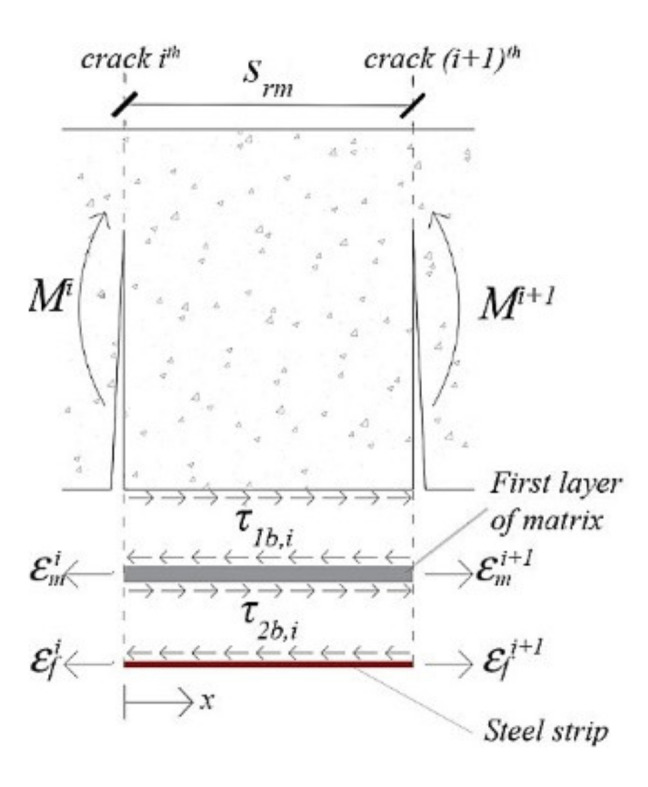
Concrete tooth between two flexural cracks.

Figure 5 shows *i*^th^ concrete tooth between two flexural cracks. The stresses in the external reinforcement are transferred by means of bond stresses *τ_2b,i_* to the inorganic matrix and then by means of bond stresses *τ_1b,i_* to the concrete substrate. $\varepsilon_{f}^{i+1}$, $\varepsilon_{m}^{i+1}$and $\varepsilon_{f}^{i}$, $\varepsilon_{m}^{i}$ are the strengthening strip and matrix strains at (*i*+1)^th^ and *i*^th^ crack, respectively.

With reference to concrete–matrix interface (Figure 5), the bond stress at *i*^th^ concrete tooth is equal to the difference of the steel strip force between two flexural cracks divided by the bonding area between them:

(18) $$\tau_{1b,i}=\frac{E_{f}b_{f}t_{f}\left(\varepsilon_{f}^{i+1}-\varepsilon_{f}^{i}\right)}{b_{f}s_{rm}}$$

The equation above defines a mean global value for shear stress that results from a change in bending moment along the beam. The stress limitation (*τ_lim_*) at concrete–matrix interface is assumed different for EB and IRS techniques. The experimental data [16,17] highlight that for EB-SRGM system the debonding occurs without damage at concrete substrate and consequently the bond strength of the external reinforcement is assumed as the stress limitation (*τ_lim_ = τ_fm_*) [3]. Instead, with the IRS-SRGM system, the strength of matrix–concrete interface is better than the EB technique due to the superior adhesion of the inorganic matrix to the concrete substrate, ensured by the application of the matrix directly on the surface of the transverse and longitudinal steel bars [16,17]. Therefore, for IRS-SRGM system, the stress limitation is assumed to be equal to the bond shear strength of concrete [3]. Adopting the Mohr–Coulomb failure criterion, in the case of zero normal stress the bond shear strength of concrete can be assumed equals 1.8 times the tensile strength. It should be noted that, similar to FRP systems, the debonding at concrete–matrix interface depends on the properties of concrete substrate, which is the weakest link of the interface.

Two different safety factors for intermediate debonding at concrete–matrix interface, $SF_{ID,EB}^{i}$ and $SF_{ID,IRS}^{i}$, are computed for the EB and IRS systems, respectively.

(19) $$SF_{ID,EB}^{i}=\frac{\tau_{1b,i}}{0.44f_{tu}}\leq 1$$

(20) $$SF_{ID,IRS}^{i}=\frac{\tau_{1b,i}}{1.8f_{tu}}\leq 1$$

The values of $SF_{ID,EB}^{i}$ and $SF_{ID,IRS}^{i}$ are checked for each load step at each concrete tooth between two cracks. If $SF_{ID,EB}^{i}$ or $SF_{ID,IRS}^{i}$ is greater than one, intermediate debonding failure at concrete–matrix interface occurs and the analysis is stopped.

With reference to steel strip–matrix interface, it assumes that in the cracked regions of EB/IRS strengthened RC beams, the interface behaviour between external reinforcement and *i*^th^ concrete tooth (Figure 5) is similar to that of SRGM–concrete joints, in which the steel strip is pulled from both ends. Considering the reference system shown in Figure 5, the distribution of interfacial stresses *τ_2b,i_* is determined by solving the following differential equation:

(21) $$\frac{d^{2}s_{i}(x)}{dx^{2}}=\frac{\tau_{bond}(x)}{E_{f}t_{f}}$$

where *s_i_ (x)* is the steel strip–matrix slip at *i*^th^ concrete tooth. Equation (21) is obtained considering the equilibrium in the horizontal direction at steel strip–matrix interface and assuming that the concrete substrate is rigid compared with external strengthening layer (*ε_f_ (x) = ds (x)/dx*). By substituting the expression of local bond–slip law (Equation (14)), Equation (21) gives:

(22) $$\frac{d^{2}s_{i}(x)}{dx^{2}}=A^{2}B\exp\left(-Bs_{i}(x)\right)\left[1-\exp\left(-Bs_{i}(x)\right)\right]$$

**Figure 6:**
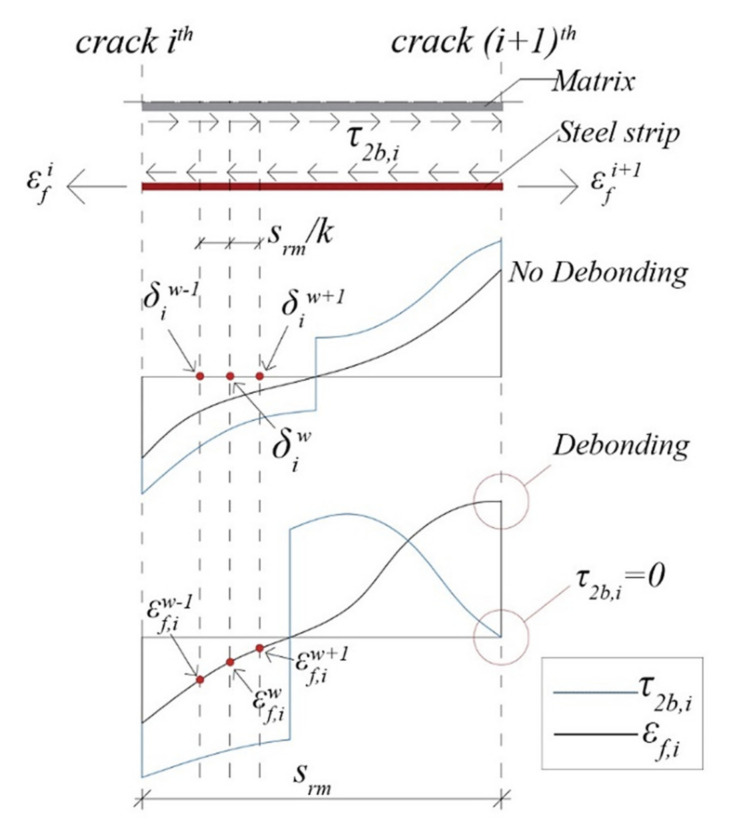
Steel strip–matrix interface at *i*^th^ concrete tooth.

Equation (22) is integrated using the finite difference method, dividing *s_rm_* into *k* (=150) segments (Figure 6). To solve the boundary value problem, the following boundary conditions at each concrete tooth are enforced:$\varepsilon_{f}(x=0)=\varepsilon_{f}^{i}$ and $\varepsilon_{f}(x=s_{rm})=\varepsilon_{f}^{i+1}$. As a result, for each load step, slips at steel strip–matrix interface and strains along the strengthening strip are obtained. By knowing the steel strip strains in *i*^th^ concrete tooth, bond stress $\tau_{2b,i}^{w}$ at *w*^th^ segment is calculated as follows (Figure 6):

(23) $$\tau_{2b,i}^{w}=E_{f}t_{f}\frac{d\varepsilon_{f,i}^{}(x)}{dx}=E_{f}t_{f}\frac{\left(\varepsilon_{f,i}^{w+1}-\varepsilon_{f,i}^{w}\right)}{\delta_{i}^{w+1}-\delta_{i}^{w}}$$

where $\varepsilon_{f,i}^{w+1}$, $\varepsilon_{f,i}^{w}$ and$\delta_{i}^{w+1}$, $\delta_{i}^{w}$ are the steel strip strains and positions at (*w + 1*)^th^ and *w*^th^ segment in *i*^th^ concrete tooth ($\delta_{i}^{w+1}-\delta_{i}^{w}$*= s_rm_/k*), respectively.

The debonding occurs when there is no strain variation in the strengthening strip and then the bond stress at interface is about zero. In the proposed procedure, safety factor for intermediate debonding at steel strip-matrix interface (*SF_ID,SM_*) of *i*^th^ concrete tooth is computed as follows:

(24) $$SF_{ID,SM}^{i}=\frac{0.01}{\underset{w}{\min}\left(\tau_{2b,i}^{w}\right)}\leq 1$$

If the bond stress $\tau_{2b,i}^{w}$ is lower than the tolerance value (assumed equal to 0.01), intermediate debonding failure at steel strip–matrix interface occurs, and the analysis is stopped.

The procedure presented above, for predicting debonding at steel strip–matrix interface, is similar to approach 2 proposed by Fib 14 [3] for FRP systems. In fact, the aim of approach 2 is to calculate the maximum possible increase in tensile stress (*max*Δ*σ_f_*) within the strengthening system, which can be transferred by means of bond stresses between two subsequent flexural cracks. This increase can be compared to the actual increase, due to the loading condition, calculated assuming full composite action (Δ*σ_f_*). The model assumes that debonding occurs if Δ*σ_f_ > max*Δ*σ_f_*. However, the simplified relationships proposed in Fib 14 [3] to evaluate *max*Δ*σ_f_* were derived assuming a bi-linear bond–slip law at FRP–concrete interface. This assumption is not consistent with SRGM exponential bond–slip law, presented in Section 2.3.1. Nevertheless, theoretical predictions according to approach 2 [3] are also calculated for comparison purposes. To this end, the exponential bond–slip law is transformed in an equivalent bi-linear bond slip law with equal values of fracture energy (*G_f_* = 0.54 N/mm), maximum bond shear stress (*τ_max_* = 1.76 MPa) and corresponding slip (*s_max_* = 0.12 mm). Furthermore, approach 2 [3] assumes that no debonding can occur in the constant bending span. In fact, under constant bending moment, there is no strain variation in the external reinforcement and consequently *Δσ_f_* = 0 (*maxΔσ_f_* ≥ 0). The experimental studies highlight that in many beams tested under four-point bending, intermediate debonding at steel strip–matrix interface in constant bending span occurs before the debonding in constant shear span.

Finally, it should be noted that the debonding at the interface between external strengthening strip and matrix is a typical failure mode of inorganic-based composite systems. In fact, all the analytical models available in literature for FRP systems [1,2,3] do not consider slippages at the fibre–matrix (epoxy resin) interface because this failure does not occur on FRP-strengthened members. However, it is predominant on RC members strengthened with inorganic-based systems, as also confirmed by single-lap shear bond tests (Section 2.3.1).

#### 2.3.4. Flowchart of the Standard Procedure

**Figure 7:**
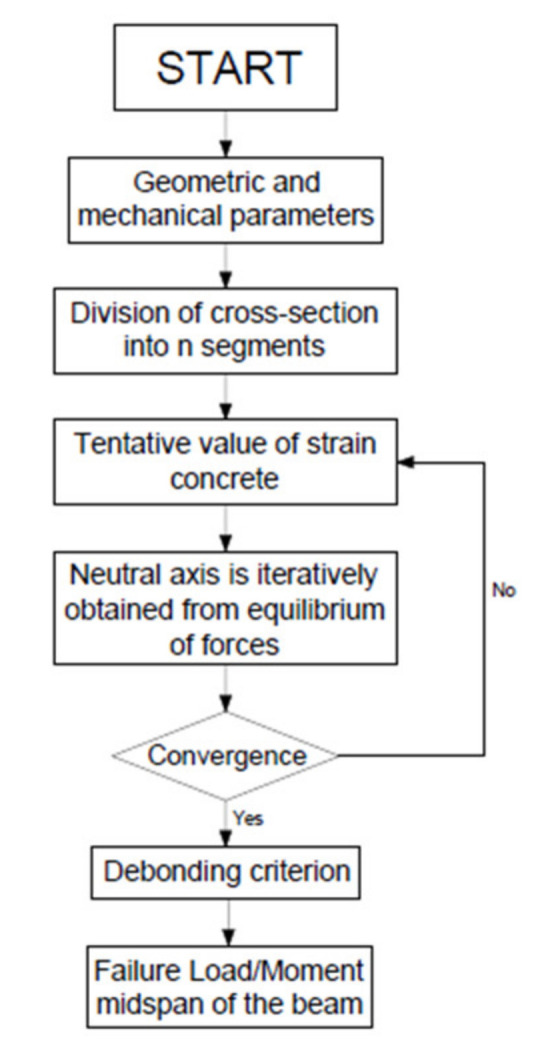
Flowchart of the numerical procedure.

In order to have a better understanding of the algorithm used in the analysis the flowchart of the code developed is presented in Figure 7.

## 3. RC Beams Strengthened with EB/IRS-SRGM

**Figure 8:**
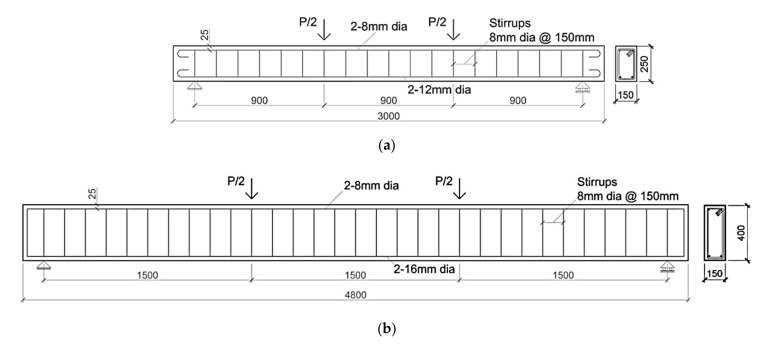
Geometrical details of the RC beams: (**a**) Group A; (**b**) Group B.

To properly implement the numerical procedure, the first experimental work considered in this study refers to a specific set of RC beams strengthened with EB-SRGM and IRS-SRGM systems tested by Bencardino and Condello [16,17]. The geometrical and mechanical parameters required for the simulations are provided below. Two groups of beams (A and B) were tested. Each group included three RC beams: one strengthened with the IRS-SRGM system (A-IRS, B-IRS), one externally strengthened with the EB-SRGM system (A-EB, B-EB) and another unstrengthened control beam (A-CB, B-CB). The geometrical details of the RC beams are shown in Figure 8.

To simulate a wide range of existing RC structures built in the 1960s and 1970s, the specimens were made using low concrete strength and both corroded smooth (12 mm diameter bars) and ribbed steel bars (8 mm and 16 mm diameters). The average values of the material properties were: concrete compressive strength of 16.8 MPa, concrete tensile strength of 1.7 MPa, steel yielding strength of 543.8 MPa, 367.1 MPa and 492.0 MPa for the 8 mm, 12 mm, and 16 mm diameters, respectively. The external strengthening system included a unidirectional reinforcing strip, made of stainless-steel cords (elastic modulus, *E_f_* = 188,360 MPa; ultimate tensile strength, *f_fu_* = 1470 MPa; equivalent thickness, *t_f_* = 0.24 mm), embedded in a polymer-based inorganic matrix. By using both the EB and IRS techniques, the RC beams were strengthened with a single layer of stainless-steel strip having *b_f_ x l_f_* (*b_f_* and *l_f_* are the width and length of the reinforcing strips, respectively) equal to 100 mm × 2600 mm and 150 mm × 4400 mm for groups A and B, respectively. Complete details of the experimental investigation including capacity, ductility and failure modes, can be found in Bencardino and Condello [16,17].

## 4. Validation of the Theoretical Procedure

In order to validate the proposed model, experimental data available in the literature related to nineteen RC beams strengthened with SRGM or similar (SRG) systems were collected. The experimental works show that the failure modes of SRG-strengthened RC beams [21,22,23] are similar to SRGM strengthened RC beams [16,17,19], i.e., debonding at concrete–matrix and/or steel strip–matrix interfaces without damage at the concrete substrate.

The main geometrical and mechanical properties of the specimens are given in Table 1. All beams were monotonically tested under four-point bending. Specifically, besides work carried out by Bencardino and Condello [16,17] presented in Section 3, the following experimental studies have been selected.

Barton et al. [21] experimentally investigated the flexural performance of four RC beams with externally bonded steel strips and organic/inorganic matrices. The variable parameters were the number of external strengthening layers as well as the type of matrix.

Pecce et al. [24] tested seven shallow RC beams strengthened with SRG and steel reinforced polymer (SRP) systems and compared them with other two shallow RC beams strengthened with FRP systems. Type of matrix (organic or inorganic), number of external strengthening layers, width of reinforcing strips and the use, or not, of nails were variable parameters.

Menna et al. [19] investigated the effectiveness of two different fibre reinforced geopolymer-based systems in strengthening of shallow RC beams. In particular, steel cord and carbon fibre reinforcing fabrics were used. Two beams for each external strengthening system were tested as well as one un-strengthened RC beam. A significant increase in the failure strength of the RC beams was experienced in case of steel cord reinforcement.

Bencardino and Condello [22] analysed the flexural behaviour of four RC beams strengthened with SRG and SRP systems. Test parameters included the use, or not, of external U-wrap end anchorages to prevent/delay delamination premature failure of the longitudinal strip.

Napoli and Realfonzo [23] tested ten RC slabs strengthened with SRG/SRP systems. The number of layers and density of the steel tape were variable parameters. Test results verified the good performance exhibited by RC slabs strengthened with the SRG system, which have shown strength increases comparable to those obtained from corresponding specimens strengthened with the SRP system.

The comparisons between theoretical results and experimental data, in terms of failure mode and ultimate load value, are given in Table 2. The experimentally observed debonding failure modes are theoretically and correctly predicted by the proposed model for 74% of the beams analysed as indicated in Table 2. It should be noted that the experimental failure mode is reported as indicated and visually observed by the authors.

With reference to intermediate debonding failure (Table 2), the numerical prediction was debonding at the concrete–matrix interface only for beam A-EB and debonding at steel strip–matrix interface for other beams (A-IRS, SRG-1, SRG-2, B-2, B-3, B-4, S(1), S(2), G1, G1-U). This result is mainly due to the value of concrete tensile strength (*f_tu_*) that affected the bond shear strength of concrete–matrix interface, calculated according to Equations (19) and (20). In fact, the beam A-EB had the lowest concrete tensile strength (Table 1), hence, intermediate debonding at concrete–matrix interface (*τ_lim_ =* 0.75 MPa) occurred before the debonding at steel strip–matrix interface, which is not affected by the properties of the concrete substrate (Equation (24)). This highlights the role of the concrete substrate on the structural effectiveness of the external strengthening system applied in the EB technique. Further experimental and theoretical research is needed on this topic. The average and standard deviation of the ratio between the proposed model and experimental failure loads are 1.02 and 7% (Table 2), respectively, highlighting the accuracy of the proposed procedure.

## 5. Theoretical Results and Comparisons

**Figure 9:**
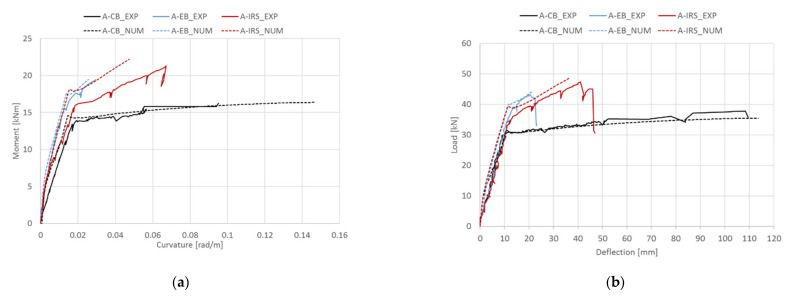
Experimental/theoretical comparisons (group A): (**a**) moment–curvature; (**b**) load–midspan deflection.

**Figure 10:**
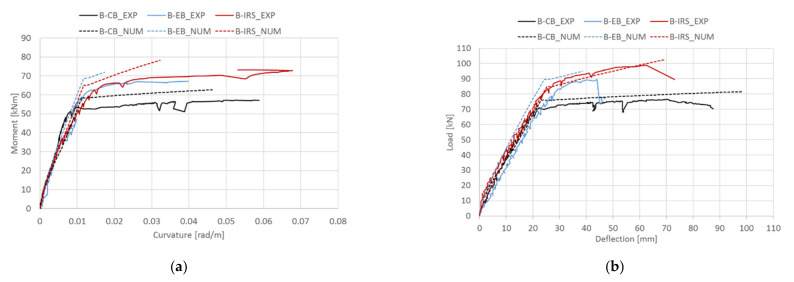
Experimental/theoretical comparisons (group B): (**a**) moment–curvature; (**b**) load–midspan deflection.

The reliability of the proposed procedure is further examined by comparing theoretical results with other experimental results, including curvature and deflection. Figure 9a,b show, for the beams of group A presented in Section 3, the experimental/theoretical moment–curvature and load–midspan deflection curves, respectively. Similar comparisons for the beams of group B are also shown in Figure 10a,b, respectively. In general, the theoretical predictions in terms of load–midspan deflection curves fit well with the experimental ones but are slightly stiffer. This is probably due to the assumption of perfect bond between internal steel bars and the concrete, and SRGM system–concrete substrate in the strengthened RC cross-section analysis. However, the error is negligible. The moment–curvature relationships (Figure 9a and Figure 10a) show less agreement with the experimental results. This can be attributed to the assumption of a perfect bond which underestimates the deformations due to the slip that occurs between the composite material/concrete and internal steel bars/concrete.

Table 3 summarizes, for the beams presented in Section 3, the theoretical and experimental values of debonding/ultimate load. The theoretical values are calculated by subtracting the effects of the beam self-weight and weight of the experimental set-up. It refers to the three debonding failures computed according to the proposed procedure as well as approach 2 [3]. For each strengthened RC beam, the failure load is evaluated as the minimum of the three debonding loads excluding approach 2, reported only as comparison. In this regard, it should be noted that the predictions obtained according to approach 2 are, for all strengthened beams, lower than the corresponding values calculated by the integration of the differential equation at each pair of flexural cracks (Int. deb. Fibre–matrix). This result could be due to the shape of the bond–slip law adopted in the analysis. In fact, although the interfacial parameters are equal for both models, the exponential bond–slip law used in the proposed procedure provides higher ultimate slip at interface compared with the equivalent bi-linear bond–slip law used in approach 2.

**Figure 11:**
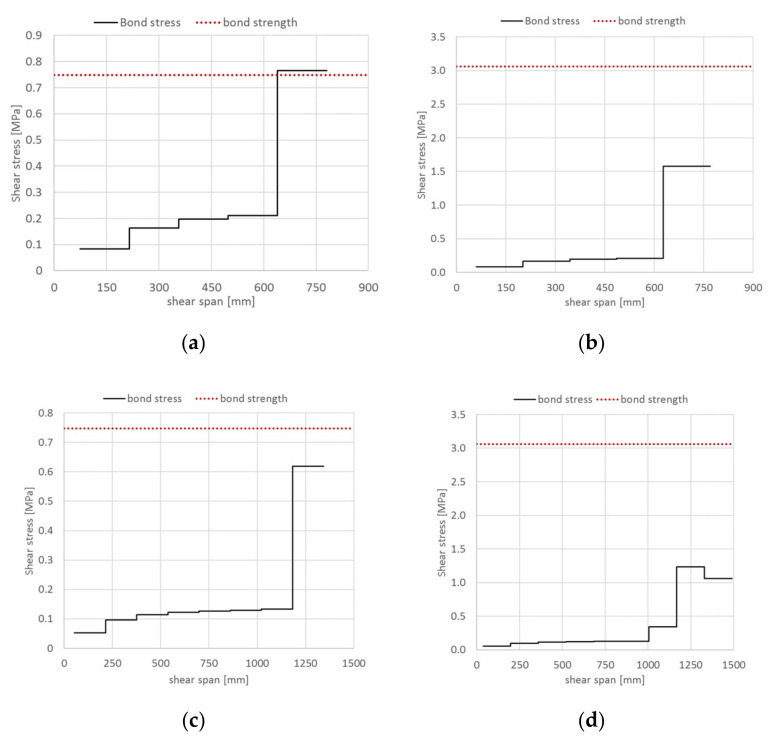
Intermediate debonding prediction at concrete–matrix interface (shear span): (**a**) beam A-EB; (**b**) beam A-IRS; (**c**) beam B-EB; (**d**) beam B-IRS.

Figure 11 shows, for all strengthened beams, the bond stresses at concrete–matrix interface in shear span at failure/debonding load (Table 3). It should be noted that for beam A-EB, the bond stresses exceed the corresponding bond strength near the loading point. As a result, beam A-EB failed by brittle debonding at concrete–matrix interface (Equation (19)).

**Figure 12:**
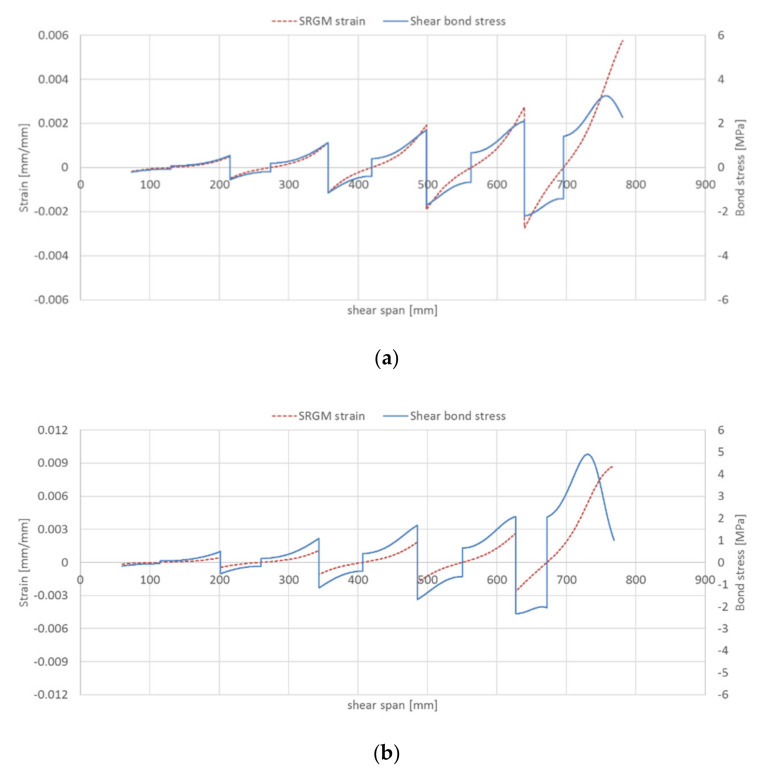

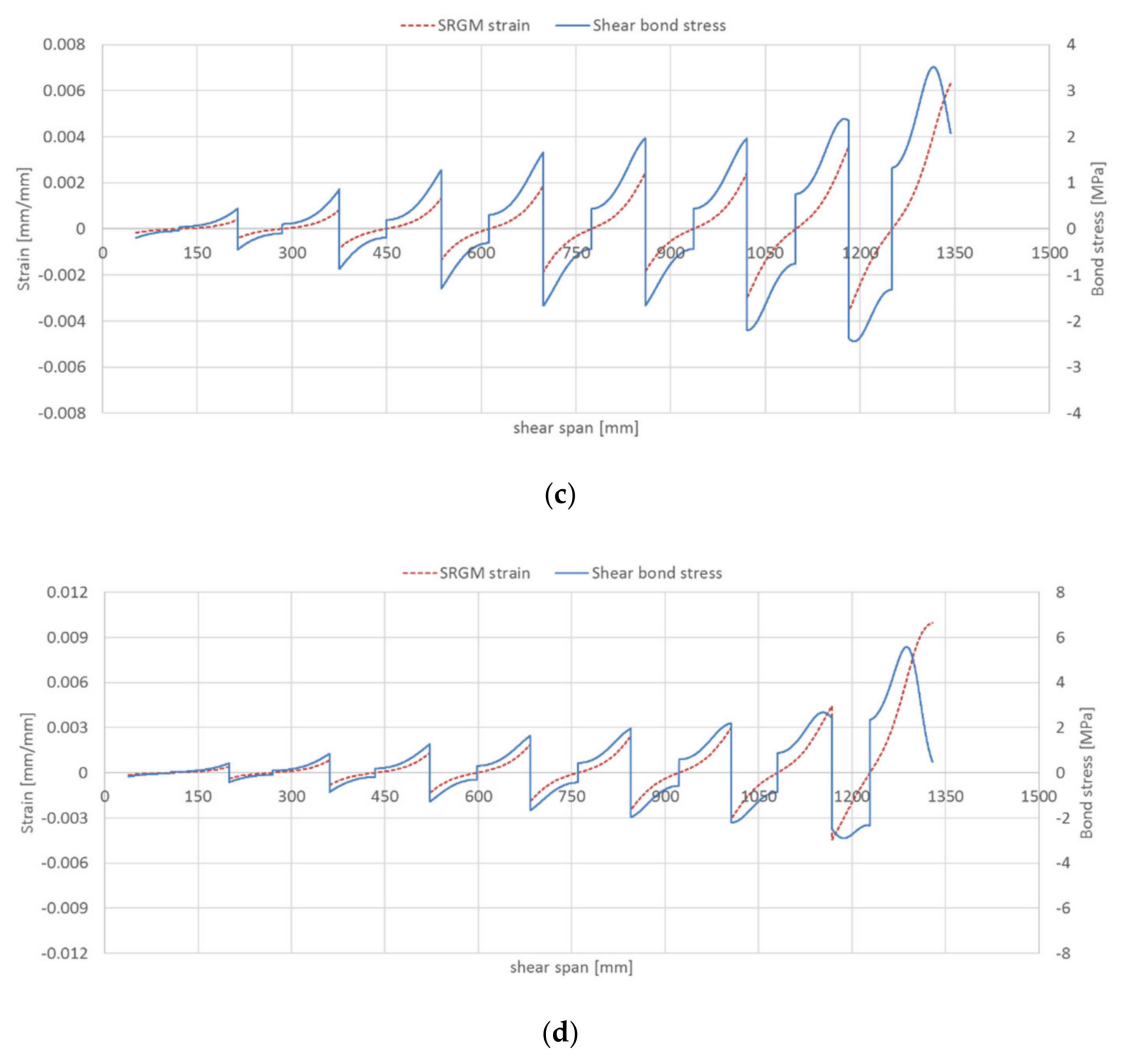
Intermediate debonding prediction at steel strip–matrix interface (shear span): (**a**) beam A-EB; (**b**) beam A-IRS; (**c**) beam B-EB; (**d**) beam B-IRS.

**Figure 13:**
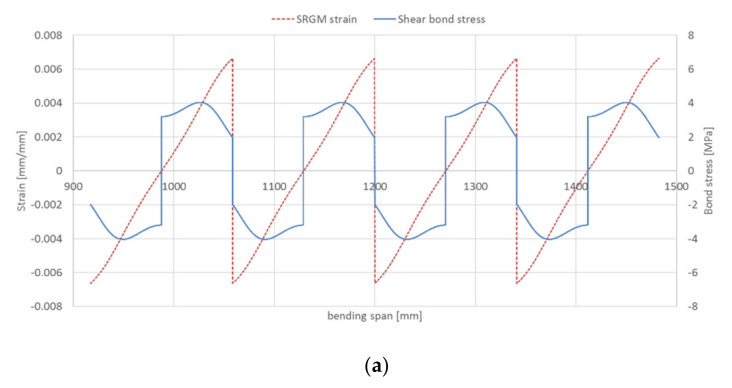

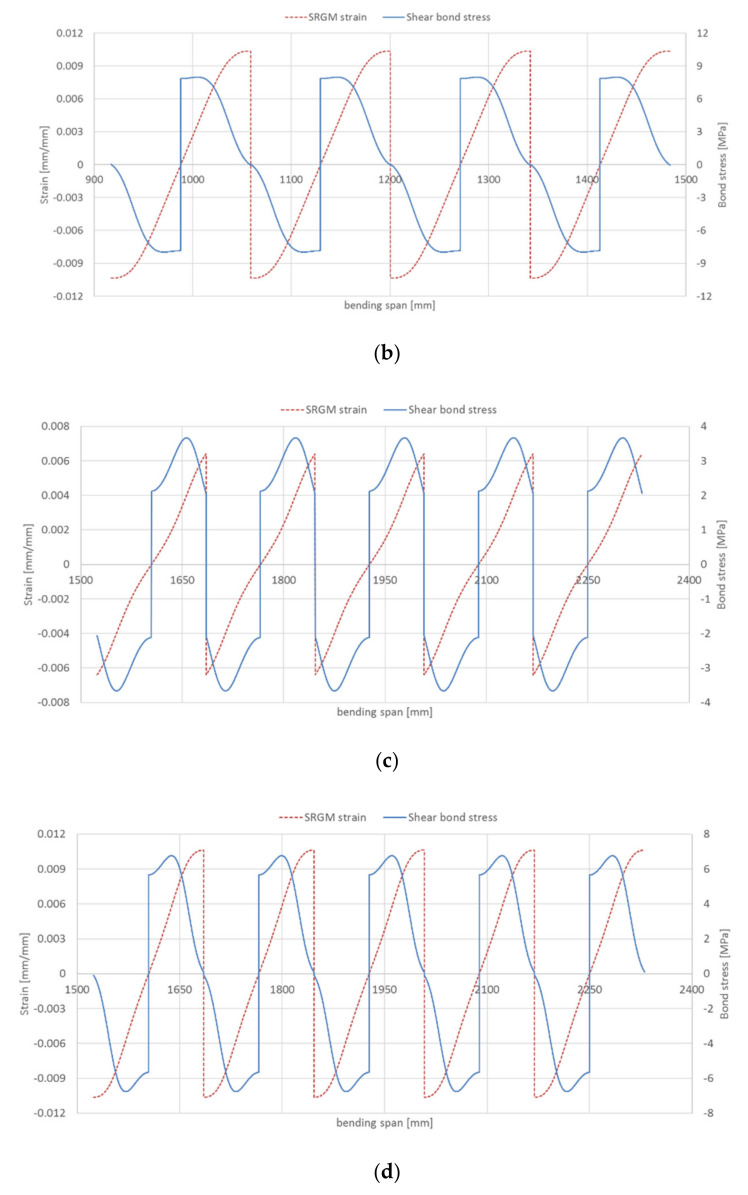
Intermediate debonding prediction at steel strip–matrix interface (half bending span): (**a**) beam A-EB; (**b**) beam A-IRS; (**c**) beam B-EB; (**d**) beam B-IRS.

As regards to steel strip–matrix interface, the bond stresses/SRGM strains for all strengthened beams (groups A and B) in the shear and half bending spans are given in Figure 12 and Figure 13, respectively. These Figures highlight that, for the beams strengthened with IRS technique (A-IRS, B-IRS), the debonding at steel strip–matrix interface occurs in the bending span. In fact, Figure 13b,d show that the shear bond stresses, at the ends of each concrete tooth, is about zero (Equation (24)). Note that interface cracking progresses from the two ends towards the midpoint of concrete tooth. For the other strengthened beams, both in shear and bending spans, bond stresses at steel strip–matrix interface are different to zero and then interlaminar debonding does not occur. Figure 13b,d point out that the maximum axial strain in external reinforcing strip, for intermediate debonding at stainless steel–matrix interface, is about 1.30–1.40 times the end debonding strain (*A =* 7.82‰). This is mainly due to the interaction between the two ends of the strengthening strip (at midpoint of each concrete tooth, both bond stress and force are non-zero), because the available bonded length is insufficient to equilibrate the applied force (*l_eff_ > s_rm_/2*) and therefore the two parts “hook” to each other.

In general, the failure modes predicted by the proposed numerical technique are consistent with experimental ones. In fact, the unstrengthened RC beams failed, both experimental and theoretical, by concrete crushing. The RC beams strengthened with EB-SRGM system failed experimentally by end debonding, whereas numerical predictions were end debonding for beam B-EB and intermediate debonding at concrete–matrix interface for beam A-EB. Beams A-IRS and B-IRS failed experimentally by intermediate debonding at steel strip–matrix interface and concrete crushing, respectively; whereas, the theoretical failure modes were debonding at steel strip–matrix interface for both strengthened beams.

However, for beam B-IRS, the theoretical strain at concrete extreme compression fibre was 3.67‰. This value is higher than the conventional ultimate strain suggested by the main standards/codes (for example, considering an equivalent stress block, *ε_cu_* is equal to 0.0035 and 0.003 according to Eurocode 2 [38] and ACI 318-19 [44], respectively) because the model adopted in the analysis to simulate the uniaxial compressive behaviour of concrete is defined by a nonlinear law with softening (Figure 1a).

## 6. Conclusions

An iterative method for predicting the flexural behaviour of RC beams externally strengthened with steel strips and inorganic matrices (EB/IRS-SRGM, EB-SRG systems) has been developed and presented. The moment–curvature relationship is numerically obtained by considering force equilibrium and strain compatibility. The beam deflection is then calculated from the midspan curvature. Failure modes and loads involving end debonding and/or intermediate debonding at steel strip–matrix and concrete–matrix interfaces are also predicted.

The accuracy of the model is examined by comparing theoretical results with experimental data available in literature. A reasonable agreement between the failure modes and failure loads from the current analysis and those from experiments is achieved. Based on the results obtained some concluding remarks can be drawn:

- The end debonding failure, for bonded lengths equal to or greater than effective bonded length, can be predicted by limiting the strain in the external strengthening strip, at first flexural crack, to the maximum analytical strain calibrated on single-lap shear bond tests. For bonded lengths lower than the effective bonded length a parabolic relationship can be used for reducing the maximum strain.
- The maximum strain value in the strengthening system for intermediate debonding at steel strip–matrix interface is about 1.30–1.40 times the end debonding strain (7.82‰). As a result, the load carrying capacity of strengthened RC beams for debonding failure at fibre–matrix interface can be predicted by limiting the average axial strain in the strengthening strip to 10–11‰.
- The brittle intermediate debonding at concrete–matrix interface can be predicted by limiting the maximum shear bond stress to the interface shear strength.
- Approach 2 proposed by Fib 14 for FRP systems provides conservative values of failure load compared with the values calculated according to the proposed model.
- The assumption of a perfect bond between materials leads to slightly stiffer behaviour of the numerical curves in the first stage (until the yielding of the internal tensile reinforcements). In any case, the yielding and failure loads are accurately predicted by the calculation procedure. Further development of the code could take into account a suitable bond–slip law between reinforcing steel bars/concrete and external composite material/concrete.

The developed model is a useful tool for the prediction of structural behaviour of RC beams strengthened with steel strips embedded into inorganic matrices. Furthermore, varying the interface bond–slip law, the procedure can be adjusted to predict the flexural response of RC beams externally strengthened with other composite materials, both inorganic and organic based systems. This topic needs to be addressed in future works.

## Figures and Tables

**Table 1:** Experimental database.

Experimental Work	Specimen	Dimensions (*b* × *h* × *l*)	*f_c_’* (MPa)	*f_tu_* (MPa)	*f_sy_* (MPa)	*A_s_* (mm^2^)	*A_f_* (mm^2^)	*E_f_* (MPa)	*ε_fu_*	*b_f_/b_c_*	N. Plies	Anchorage System
Bencardino and Condello [16,17]	A-EB	150 × 250 × 3000	16.80	1.70	367.10	226.08	24.00	188,360	0.02	0.67	1	-
	A-IRS											
	B-EB	150 × 400 × 4500			492.00	401.92	36.00			1.00		
	B-IRS											
Barton et al. [21]	SRG-1	203 × 305 × 2438	36.50	3.36	436.00	401.92	57.76	206,000	0.02	0.75	1	-
	SRG-2						115.52				2	
Pecce et al. [24]	B-2	400 × 200 × 3700	33.30	3.10	500.00	392.50	54.00	184,000	0.02	0.50	1	-
	B-3									0.50	1	Nails
	B-4						108.00			0.50	2	Nails
Menna et al. [19]	S(1)	400 × 200 × 3800	25.00	2.38	450.00	392.50	72.00	210,000	0.01	0.50	1	-
	S(2)											-
Bencardino and Condello [22]	G1	150 × 250 × 3000	34.00	3.50	604.20	157.00	28.50	206,000	0.02	1.00	1	-
	G1-U											U-wrap
Napoli and Realfonzo [23]	SRG-1LD	400 × 200 × 3700	15.14	2.60	460.00	392.50	16.80	206,000	0.02	0.50	1	-
	SRG-2LD						33.60				2	-
	SRG-1MD						50.80				1	-
	SRG-1MD-A										1	-
	SRG-1MD-B										1	-
	SRG-2MD						101.60				2	-

**Table 2:** Theoretical/experimental comparisons.

Specimen	Failure Mode		Ultimate Load (kN)		*F_num_./F_exp_.*
	Experimental	Theoretical	Experimental (*F_exp_.*)	Theoretical (*F_num_.*)	
A-EB	End debonding	Int. debonding	43.22	44.29	1.02
A-IRS	Int. debonding	Int. debonding	47.39	48.53	1.02
B-EB	End debonding	End debonding	89.52	94.26	1.05
B-IRS	Concrete crushing	Concrete crushing	98.96	102.33	1.03
SRG-1	Int. debonding	Int. debonding	81.80	87.17	1.07
SRG-2	Int. debonding	Int. debonding	92.40	93.17	1.01
B-2	Int. debonding	Int. debonding	72.70	74.50	1.02
B-3	Int. debonding	Int. debonding	71.50	74.50	1.04
B-4	Int. debonding	Int. debonding	86.70	88.47	1.02
S(1)	Steel cord failure	Int. debonding	85.70	76.58	0.89
S(2)	Steel cord failure	Int. debonding	86.30	76.58	0.89
G1	Int. debonding	Int. debonding	54.93	62.44	1.14
G1-U	End-anch. deb/slip	Int. debonding	54.93	62.44	1.14
SRG-1LD	Rupture SRG	End debonding	61.85	66.45	1.07
SRG-2LD	Delam. SRG system	End debonding	68.70	67.70	0.99
SRG-1MD	Delam. SRG system	End debonding	69.55	69.12	0.99
SRG-1MD-A	Delam. SRG system	End debonding	70.25	69.12	0.98
SRG-1MD-B	Delam. SRG system	End debonding	64.28	69.12	1.08
SRG-2MD	Delam. SRG system	End debonding	87.58	77.03	0.88
				Average	1.02
				Standard deviation	0.07

**Table 3:** Debonding/Ultimate load values (kN).

Beam	Experimental (*F_exp_.*)	Theoretical				
		End Deb.	Int. Deb. Concrete–Matrix	Int. deb. Fibre–Matrix	Approach 2 Fib 14	Failure (*F_num_.*)
A-CB	37.22	-	-	-	-	35.46
A-EB	43.22	51.54	44.29	50.54	47.54	44.29
A-IRS	47.39	54.31	61.02	48.53	45.27	48.53
B-CB	76.33	-	-	-	-	81.62
B-EB	89.52	94.26	96.29	104.26	94.38	94.26
B-IRS	98.96	103.67	106.96	102.33	92.01	102.33

## Data Availability

The data presented in this study are available on request from the corresponding author.
